# Complexes with Atomic Gold Ions: Efficient Bis-Ligand Formation

**DOI:** 10.3390/molecules26123484

**Published:** 2021-06-08

**Authors:** Felix Duensing, Elisabeth Gruber, Paul Martini, Marcelo Goulart, Michael Gatchell, Bilal Rasul, Olof Echt, Fabio Zappa, Masoomeh Mahmoodi-Darian, Paul Scheier

**Affiliations:** 1 Institut für Ionenphysik und Angewandte Physik, Universität Innsbruck, Technikerstr. 25, A-6020 Innsbruck, Austria; felix.duensing@uibk.ac.at (F.D.); E.Gruber@uibk.ac.at (E.G.); Paul.martini@fysik.su.se (P.M.); m.goulart@rug.nl (M.G.); michael.gatchell@uibk.ac.at (M.G.); olof.echt@unh.edu (O.E.); Fabio.Zappa@uibk.ac.at (F.Z.); Paul.Scheier@uibk.ac.at (P.S.); 2 Department of Physics, Stockholm University, 106 91 Stockholm, Sweden; 3 Zernike Institute for Advanced Materials, University of Groningen, 9747 AG Groningen, The Netherlands; 4 Department of Physics, University of Sargodha, Sargodha 40100, Pakistan; bilal.rasul@uos.edu.pk; 5 Department of Physics, University of New Hampshire, Durham, NH 03824, USA; 6 Department of Physics, Karaj Branch, Islamic Azad University, Karaj 3149968111, Iran

**Keywords:** gold complexes, clusters, ligands, mass spectrometry

## Abstract

Complexes of atomic gold with a variety of ligands have been formed by passing helium nanodroplets (HNDs) through two pickup cells containing gold vapor and the vapor of another dopant, namely a rare gas, a diatomic molecule (H_2_, N_2_, O_2_, I_2_, P_2_), or various polyatomic molecules (H_2_O, CO_2_, SF_6_, C_6_H_6_, adamantane, imidazole, dicyclopentadiene, and fullerene). The doped HNDs were irradiated by electrons; ensuing cations were identified in a high-resolution mass spectrometer. Anions were detected for benzene, dicyclopentadiene, and fullerene. For most ligands L, the abundance distribution of AuL*_n_*^+^ versus size *n* displays a remarkable enhancement at *n* = 2. The propensity towards bis-ligand formation is attributed to the formation of covalent bonds in Au^+^L_2_ which adopt a dumbbell structure, L-Au^+^-L, as previously found for L = Xe and C_60_. Another interesting observation is the effect of gold on the degree of ionization-induced intramolecular fragmentation. For most systems gold enhances the fragmentation, i.e., intramolecular fragmentation in AuL*_n_*^+^ is larger than in pure L*_n_*^+^. Hydrogen, on the other hand, behaves differently, as intramolecular fragmentation in Au(H_2_)*_n_*^+^ is weaker than in pure (H_2_)*_n_*^+^ by an order of magnitude.

## 1. Introduction

Gold has fascinated mankind for several thousand years [1]. In its bulk form it exhibits a distinct yellow color which is considerably different from most other metals that reflect the complete visible range of light and have a shiny white appearance. This yellow color is the result of a relativistic contraction of the 6s orbital [2]. This also leads to an exceptionally high atomic ionization energy for a metal of 9.225 eV [3], a large electron affinity of 2.3 eV [4] and it explains the shorter and stronger covalent bonds of gold which makes it often behave like a heavy hydrogen atom.

The subsequent chemical reactivity of gold atoms or the cations might be unexpected for a metal that does not exhibit corrosion. Kapur and Müller reported a 5% yield of NeAu^+^ upon slow field evaporation of gold in a mixture of hydrogen and neon [5]. Almost two decades later, Pyykkö calculated binding energies between Au^+^ and several ligands, including all rare gas atoms [6]. Schröder et al. determined the binding energy of XeAu^+^ by refined theoretical means to be 1.31 eV and suggested to use this value as an absolute reference for a relative gold(I) cation affinity scale [7]. In the low-pressure regime of a Fourier transform ion cyclotron resonance mass spectrometer, radiative association between Au^+^ and C_6_F_6_ leads to a weakly bound complex that was used by Schröder et al. as a precursor to generate other complexes by ligand-exchange reactions. In combined experimental and theoretical studies, they determined the binding energy of H_2_O to Au^+^ with 1.56 eV [7] and benzene with 3.04 eV [8].

The ligand most weakly bound to gold is helium [9] and thus ligand-exchange with every other ligand can be expected inside a helium nanodroplet (HND). In the case of neutral helium droplets, this was successfully demonstrated by the growth of pristine and mixed gold clusters containing a few gold atoms [10,11,12,13,14,15,16,17,18], as well as nanoparticles and nanowires that contain many thousand atoms [19,20,21,22,23,24,25]. Recently, we doped neutral HNDs containing about 10^6^ He atoms with gold vapor and investigated low-mass ions formed upon electron ionization and ejected from the massive droplets [12]. Pickup of dopants into HNDs is solely determined by the geometric cross section of the HNDs and the dopant. The kinetic energy released into a HND by the inelastic collision with a gold atom from a vapor at a temperature of 1300 K is below 0.5 eV [10], and the binding energy released upon gold cluster formation is between 2.6 and 4.7 eV [26]. The sum of these energies is released into the helium matrix and leads to the evaporation of about 1600 He atoms per eV [27]. For droplets that contain He atoms in the order of a million, even the formation of a gold cluster consisting of several gold atoms results in negligible shrinking of the HNDs. The log-normal size distribution of the neutral HNDs in combination with Poisson pickup statistics is expected to result in a log-normal size distribution of the neutral gold clusters embedded in HNDs.

However, the mass spectra of pristine gold cluster ions formed upon electron irradiation of neutral HNDs doped with gold, exhibit pronounced intensity anomalies [10], similar to gold cluster size distributions obtained by conventional techniques [28,29]. Magic numbers and shell closures in cluster size distributions are the result of fragmentation where less stable cluster sizes are depleted and decay into more stable ones that more likely survive [30]. For heliophilic dopants such as gold, charge transfer from He^+^ or a small He*_n_*^+^ ionic core to a dopant cluster is the dominant ionization mechanism upon electron irradiation of doped HNDs at sufficiently high electron energies [27,31,32,33,34]. The high ionization energy of helium compared to all dopants makes this reaction highly exothermic and the excess energy released into the internal degrees of freedom of the ionized dopant cluster is expected to lead to fragmentation and subsequently to the observed intensity anomalies in the cluster size distributions. Tiefenthaler et al. demonstrated that pickup of gold into charged HNDs leads to gold cluster size distributions that are free of local intensity anomalies [15]. In this experiment charge transfer from helium happens to a single gold atom and the binding energy of additional gold atoms attaching to Au*_n_*^+^ (*n* ≥ 1) is quenched by the helium matrix. By using this technique, the stoichiometric cation SF_6_^+^ could be detected for the first time via pickup of SF_6_ into charged HNDs, as recently demonstrated by Albertini et al. [35]. Pre-doping of charged HNDs with hydrogen was successfully utilized to prevent fragmentation of fragile molecular dopants by a gentle proton transfer reaction and avoid the exothermic charge transfer process from He*_n_*^+^ [36,37].

Besides pristine gold cluster ions Au*_m_*^+^, binary cluster ions of the form He*_n_*Au*_m_*^+^ were also observed [12,14]. For He*_n_*Au^+^, in excess of *n* = 100 helium atoms were seen. A pronounced maximum at *n* = 12 was explained by the completion of an icosahedral shell and a local maximum at *n* = 14 by another stable structure where two additional helium atoms can be squeezed into the first shell, similar to He*_n_*Kr^+^, where also *n* = 14 turned out to be magic [38]. Pronounced magic numbers were also reported in a subsequent study where HNDs were doped with gold vapor and another rare gas Rg (Rg = Ne, Ar, Kr or Xe) [14]. For all four noble gases, the intensity of Rg_2_Au^+^ exhibits a local maximum which agrees in the case of Ar, Kr and Xe with a covalent character of the bonding [6]. In the case of He and Ne, intensity anomalies at *n* = 12 indicate an icosahedral shell closure and weak physical bonding. A clear intensity drop at Ar_6_Au^+^ suggests the closure of an octahedral solvation shell. Goulart et al. studied mixed cluster ions of gold and C_60_ [13]. For both polarities ions of the form (C_60_)_2_Au^±^ are particularly abundant. Density functional theory calculations suggest a sandwich like structure similar to LAuL^±^ ions previously reported for several smaller ligands L [39,40,41,42,43,44,45,46,47,48]. In the present study, we extend these ligand switching reactions to heavy rare gases and molecules including the diatomic species H_2_, N_2_, O_2_, P_2_, and I_2_, the molecules H_2_O, CO_2_, and SF_6_, and the carbonaceous species C_6_H_6_, C_3_H_4_N_2_, C_5_H_6_ (and its dimeric form), C_10_H_16_, and C_60_. In the case of adamantane, benzene and C_60_, anionic complexes with gold were also investigated.

## 2. Results and Discussion

### 2.1. Cations

**Figure 1:**
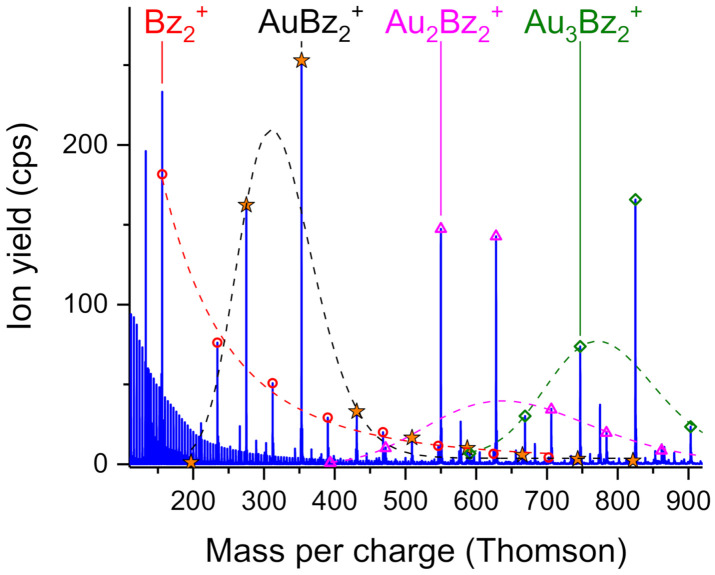
Mass spectrum of positively charged helium droplets doped with gold and benzene. The AuBz_n_^+^ progression is marked with an asterisk. Cluster ion series of benzene containing zero, two and three gold atoms are designated by red open circles, pink triangles and green diamonds, respectively. The dashed lines are log-normal peak fits to the data points, omitting exceptionally intense (magic) cluster sizes.

Figure 1 shows a mass spectrum of positively charged helium droplets doped with gold (Au, atomic mass 197) and benzene (Bz, mass 78). In the range up to 400 Thomson, the series of He*_n_*^+^ cluster can be seen. The more prominent peaks are almost exclusively combinations of gold and benzene. The pristine benzene cluster series is indicated by the open red circles; it follows a typical log-normal distribution (red dashed line), free of local intensity anomalies. Please note that the peak Bz_2_^+^ coincides with He_39_^+^ which was subtracted from the measured ion yield to obtain the correct peak height of the benzene dimer cation. The AuBz*_n_*^+^ progression is marked with an asterisk and complexes containing two or three gold atoms are designated with open pink triangles and open green diamonds, respectively. All these mixed gold-benzene cluster series exhibit pronounced intensity anomalies. The ion yields of AuBz_2_^+^, Au_2_Bz_2_^+^, Au_2_Bz_3_^+^ and Au_3_Bz_3_^+^ are too high to include them in a log-normal peak fit ($y=y_{0}+\frac{A}{\sqrt{2\pi }wx}e^{-{\left(ln\left(x/x_{c}\right)\right)}^{2}/2w^{2}}$ with *y*_0_, *A*, *x_c_* and *w* as fit parameters) of the corresponding cluster size distributions (dashed lines in respective colors).

**Figure 2:**
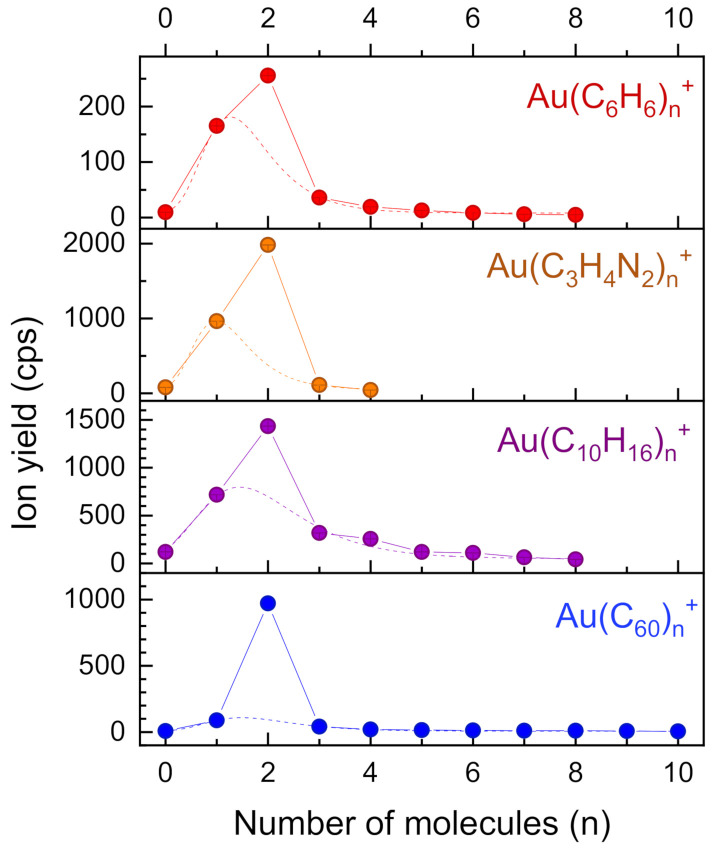
Intensity distribution of cationic complexes AuL_n_^+^ of a single gold atom and carbon containing ligands L. The dashed lines are peak functions fitted to the data excluding the value at *n* = 2.

The ion yield of AuBz_n_^+^ is plotted as a number of benzene molecules *n* in Figure 2 (C_6_H_6_, red circles). The log-normal fit to the data (not including *n* = 2) is shown as a dashed line. Cluster size distributions of several other mixed clusters of one gold atom and carbon containing molecules were obtained in a similar way for imidazole (C_3_H_4_N_2_, orange circles), adamantane (C_10_H_16_, purple) and buckminsterfullerene (C_60_, blue); the log-normal fits to the data (excluding *n* = 2) are shown as dashed lines.

All cluster size distributions in Figure 2 show a pronounced maximum at *n* = 2. As in the case of benzene, peak functions (log-normal, or extreme—$y=y_{0}+A\cdot e^{\left(-e^{-z}-z+1\right)}$. with $z=\frac{x-x_{c}}{w}$ and *y*_0_, *A*, *x_c_* and *w* as fit parameters) were fitted to the data, omitting the value for *n* = 2 (dashed lines). The enhancement of the ion yields at *n* = 2 relative to the dashed line is by far most prominent for C_60_. Goulart et al. [13] reported on an unusually high ion yield of complexes consisting of two fullerenes and one gold atom, both for cations and anions. Density functional theory calculations predict a dumbbell-like structure where two C_60_ molecules are located on opposite sides of the Au atom. The ratio of the ion yield at *n* = 2 and the value of the corresponding peak function fitted to the cluster size distribution at *n* = 2 is a measure for the enhancement of the bis-ligand structures and these values are listed in Table 1.

**Figure 3:**
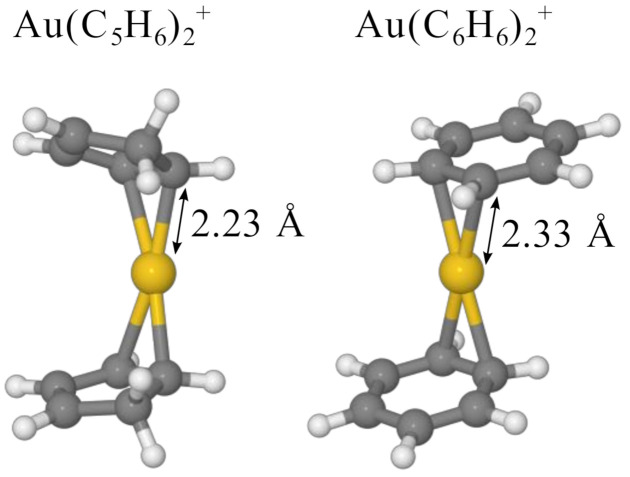
Proposed structures of Au(C_5_H_6_)_2_^+^ and Au(C_6_H_6_)_2_^+^ calculated at the M06-D3/def2-TZVP level of theory. Both systems display dumbbell-like structures that appear to be commonly formed in gold bis-ligands.

Figure 3 shows the calculated structures of Au(C_6_H_6_)_2_^+^ and Au(C_5_H_6_)_2_^+^ (the latter is from the decomposed product of di-cyclopentadiene, see below). As is seen with other bis-ligand compound containing a gold atom [11,13], the molecules display dumbbell-like structures centered around the metal. The gold cation forms covalent bonds over two adjacent carbon atoms in each ring, in contrast to the *η*^5/6^ bonds present in typical metallocene species that contain other transition metals [49,50]. The binding energies are 1.86 and 2.51 eV for the first and second C_6_H_6_ ring, respectively, while the equivalent binding energies of C_5_H_6_ rings are 2.03 and 2.15 eV, respectively. Additional ligands interact through non-bonding ion-induced dipole interactions that are significantly weaker than the covalent bonds in the mono- and bis-ligand complexes. This mimics observations with other ligands [11] where only the first two ligands are covalently bound to the central Au atom and additional adducts form weakly bound shells around the ionic core.

**Figure 4:**
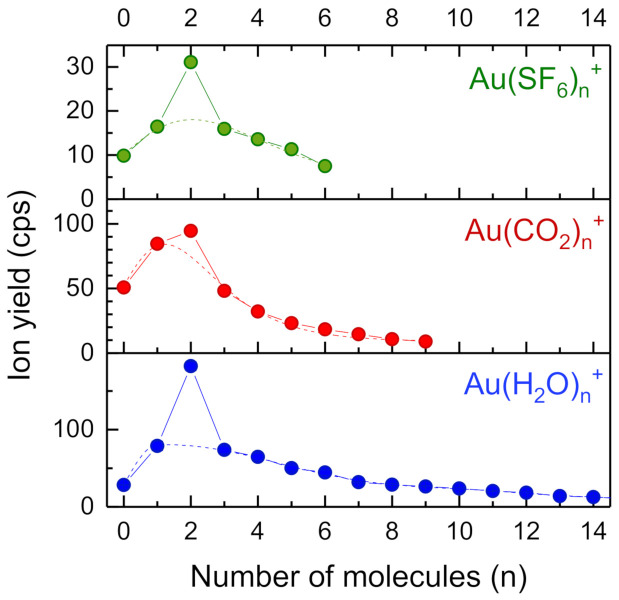
Au(SF_6_)_n_^+^, Au(CO_2_)_n_^+^ und Au(H_2_O)_n_^+^ cluster size distributions. The dashed lines are peak-fits to the data omitting the values at *n* = 2. For CO_2_ the ion yield of the bis-ligand species Au(CO_2_)_2_^+^ is only weakly enhanced.

Figure 4 shows the cluster size distributions of Au(SF_6_)*_n_*^+^, Au(CO_2_)*_n_*^+^ and Au(H_2_O)*_n_*^+^. All three molecular ligands exhibit maxima at *n* = 2. Dashed lines indicate log-normal and extreme peak fits to the data, omitting the value at *n* = 2. The deviation from these fits to the ion yield at *n* = 2 is much more pronounced for Au(SF_6_)*_n_*^+^ and Au(H_2_O)*_n_*^+^ than for Au(CO_2_)*_n_*^+^. The enhancement factors for the bis-ligand ions are listed in Table 1.

**Figure 5:**
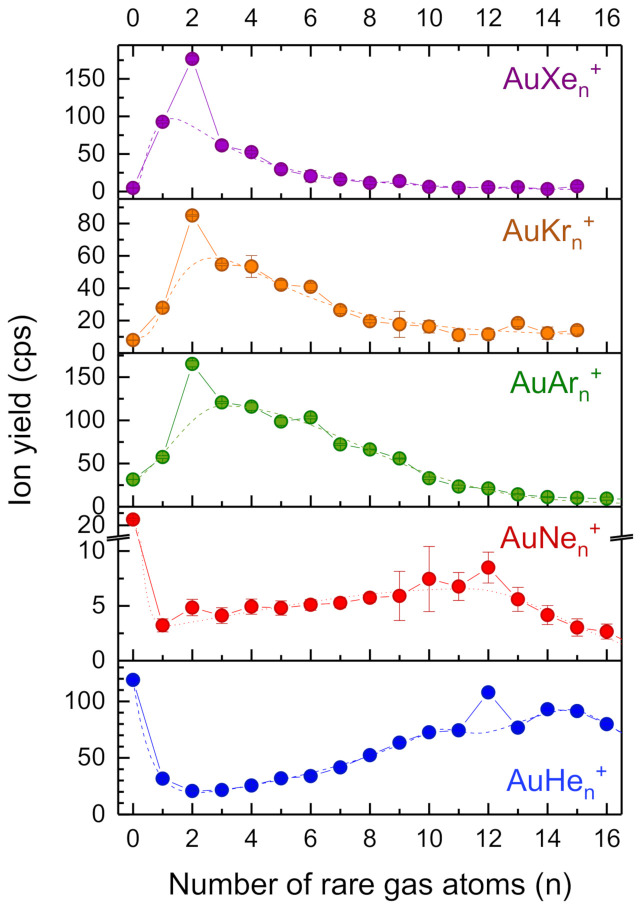
Cluster size distributions of mixed gold-rare gas complexes containing one gold atom (AuRg_n_^+^, solid circles). The dashed lines are peak fits (log-normal, extreme or bi-gaussian) to the data. In the case of Ne and He, multiple peak fits were required due to a maximum at the bare gold cation (*n* = 0). Pronounced enhancement of the bis-ligand structures is observed for Xe, Kr, and Ar, little enhancement for Ne, no enhancement for He. Additional intensity anomalies at *n* = 4, 6, and 12 indicate shell closures that can be assigned to tetrahedral, octahedral and icosahedral solvation layers, respectively [14].

Rare gas atoms (Rg) were also attached to atomic gold ions. In agreement with the strong chemical bonds calculated by Schröder et al. [7] and Pyykkö [6] for the heavy rare gas atoms, the relative yield of bare gold cations decreases with the mass of the rare gas ligand. In the case of Ne and He, Au^+^ is the most intense ion, although complexes with a larger number of rare gas ligands can be observed compared to the heavy rare gas atoms. AuRg_2_^+^ is a local maximum for all rare gases but He (see Figure 5). 

A clear local maximum appears at *n* = 12 for He and Ne, indicating an icosahedral shell closure. Ar shows two less pronounced maxima at *n* = 6 and 9 and Kr a weak one at *n* = 6. Based on a hard-sphere model a maximum at *n* = 6 can be assigned to an octahedral solvation shell [14]. The intensity drop at *n* = 4 in the case of Xe is fairly weak; it does not necessarily indicate a tetrahedral shell closure. For the heavy rare gas clusters (Ar, Kr, and Xe) a single peak function was fitted to the data, skipping the value at *n* = 2 (dashed lines). The maximum at *n* = 0 and local maxima at *n* = 12 for Ne and He required fitting with two and four bi-gaussian curves, respectively (dashed lines). Again, we calculated the enhancement factor for the bis-ligand ions as the ratio of the ion yield and the value of the dashed line at *n* = 2; results are listed in Table 1.

**Figure 6:**
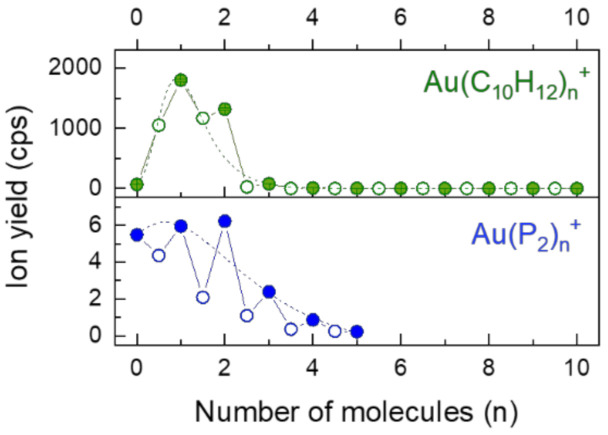
Gold ions complexed with dicyclopentadiene (C_10_H_12_, green) and phosphorus (P_2_, blue). Empty circles represent non stoichiometric ligands due to fragmentation of P_2_ into atomic phosphorus and C_10_H_12_ into C_5_H_6_.

Figure 6 shows cluster size distributions of gold cations complexed with di-cyclopentadiene (C_10_H_12_) and phosphorus as ligands. In both cases the mass spectra exhibit peaks corresponding the non-stoichiometric ligands, i.e., where one ligand is atomic phosphorus or cyclopentadiene (C_5_H_6_). Vaporizing red phosphorus at low temperatures produces a gas primarily consisting of P_4_. However, the source of phosphorus in the present study was a ceramic paste that was utilized to build the gold oven [37]. When passing HNDs through the heated oven the mass spectra revealed a long series of phosphorus cluster ions up to P_80_^+^ with stoichiometric cluster ions Au(P_2_)*_n_*^+^ strongly enhanced in intensity compared to non-stoichiometric ions AuP(P_2_)*_n_*^+^ (full and open circles in Figure 6, respectively). In the case of dicyclopentadiene thermal dissociation of C_10_H_12_ into cyclopentadiene C_5_H_6_ may occur. The presence of two different ligands may lead to three bis-ligand forms, i.e., Au(C_5_H_6_)_2_^+^, Au(C_10_H_12_)_2_^+^ and Au(C_5_H_6_)(C_10_H_12_)^+^. The high yields of ions with *n* = 1, 1.5 and 2 indicate high stability for all three bis-ligand complexes.

**Figure 7:**
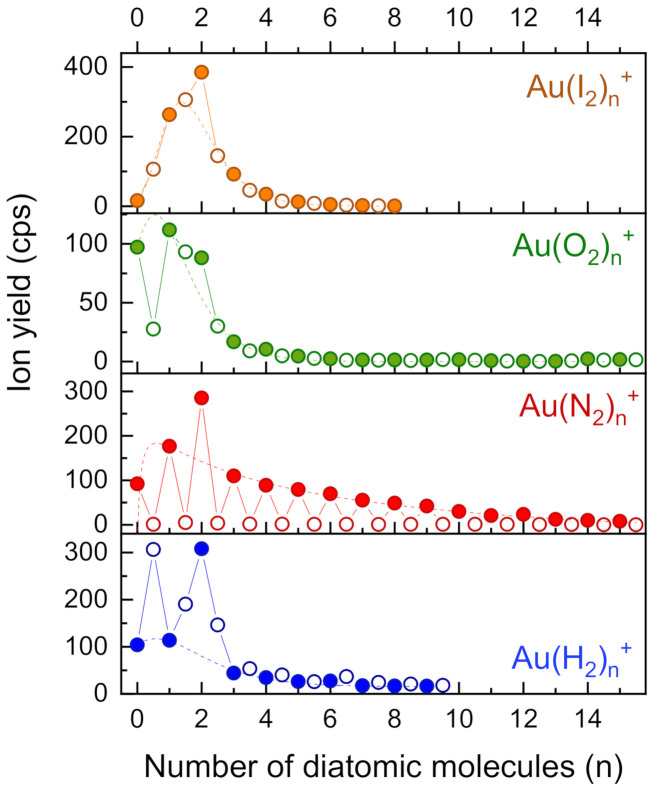
Gold ions complexed with diatomic molecules with stoichiometric (filled circles) and non-stoichiometric (empty circles) cluster progressions. The dashed lines are peak functions fitted to the stoichiometric data points, omitting *n* = 2.

Figure 7 shows cluster size distributions complexes containing one gold atom and various diatomic molecules. The gases H_2_, N_2_ and O_2_ were introduced via regulated valves into PU1; for I_2_, we slightly heated the same gas inlet. A pronounced intensity oscillation between stoichiometric and non-stoichiometric clusters is observed for complexes of Au^+^ with N_2_. The oscillation indicates little fragmentation of diatomic ligands due to the ionization process and/or chemical reactions with the gold atom. The distribution of Au complexes with I_2_, on the other hand, is entirely smooth. For the other diatomic ligands (O_2_, H_2_) the situation is intermediate (see below).

As before, we have fitted log-normal functions to the distributions of stoichiometric cluster ions, as indicated by the dashed lines in Figure 7. Again, the ions containing two molecular ligands were omitted from the fits. Enhancement factors are compiled in Table 2. All four diatomic ligands exhibit a clear enhancement at *n* = 2. Hydrogen shows a peculiarly intense Au(H_2_)_2_^+^ ion. For all other values of *n* the non-stoichiometric ions (open symbols) are dominant. In the case of oxygen, AuO^+^ is exceptionally weak. Stoichiometric clusters are slightly more intense than their non-stoichiometric neighboring species. Au(O_2_)_2_^+^ is not the most abundant ion as in all other cases, however, it still is almost a factor two higher than the value of the peak fitted to the cluster size distribution.

The ratio of the sum of all stoichiometric cluster ions and the sum of all non-stoichiometric cluster ions was determined; it is listed in Table 2. A high value indicates little fragmentation of the corresponding diatomic ligand upon ionization. In the case of nitrogen stoichiometric cluster ions are 60 times more abundant than cluster ions containing an odd number of nitrogen atoms. In the case of hydrogen, the situation is quite the opposite, i.e., the yield of non-stoichiometric ions is higher than that of stoichiometric cluster ions. The third column in Table 2, designated with Ratio pristine, was obtained from mass spectra of HNDs only doped with the corresponding ligand (without vaporizing gold in the oven).

Except for H_2_ and P_2_, all ratios are larger without gold, thus indicating enhanced fragmentation of ligands due to chemical reactions with gold. The result for phosphorous may be an artefact, because it was evaporated from the walls of the gold oven. The ratio with gold was measured at a much higher oven temperature than the ratio without gold. Consequently, thermal decomposition of P_2_ into atomic phosphorus in the vapor phase is the likely reason for the increased yield of non-stoichiometric gold-phosphorous complexes.

Hydrogen shows a remarkably different trend: Without gold, non-stoichiometric hydrogen cluster ions formed upon electron ionization of hydrogen doped HNDs are 16 times more intense than stoichiometric hydrogen cluster ions. The presence of a gold atom in the cluster ions strongly enhances (factor 16.4) stoichiometric ions. Lundberg et al. recently investigated hydrogenated gold cluster ions and observed a markedly different trend of the hydrogen attachment to odd and even numbered gold clusters [51]. According to quantum chemical calculations at the MP2/def2-TZVP level of theory, a hydrogen atom appears to bridge two Au atoms in hydrogenated cluster ions with an even number of Au atoms. In contrast, cluster ions with an odd number of gold atoms are preferentially solvated with intact H_2_ molecules in the first solvation layer. The column designated Effect of gold in Table 2 represents the increase (values >1) of fragmentation of ligands by the presence of a single gold atom in the cluster ions. Di-cyclopentadiene is most sensitive to gold and dissociated almost five times stronger. Both N_2_ and O_2_ exhibit also more fragmentation when complexed with a gold atom. P_2_ and I_2_ are almost unaffected and fragmentation of H_2_ is strongly suppressed by the presence of a gold atom in the cluster ions.

It is tempting to attribute the differences in the suppression of intramolecular fragmentation by gold on the nature of the bond between gold and the ligands. We focus on H_2_ and O_2_ for which the difference is particularly large. The interaction of O_2_ with neutral and charged gold, and its clusters, has been studied extensively because of the relevance to catalytic oxidation reactions [52,53,54]. Atomic gold, either neutral or cationic, is essentially unreactive towards O_2_. It is not surprising, then, that the presence of gold has a relatively small effect on the degree of intramolecular fragmentation (see the last column in Table 2). For hydrogen, on the other hand, the charge state of the gold atom has a large effect: Au is unreactive while Au^+^ is strongly reactive [52]. Consequently, the reaction of nascent H_2_^+^ with gold suppresses the rapid autoprotonation reaction of H_2_^+^ with H_2_ into H_3_^+^ + H that ensues upon ionization of bare (H_2_)*_n_* [55].

### 2.2. Anions

**Figure 8:**
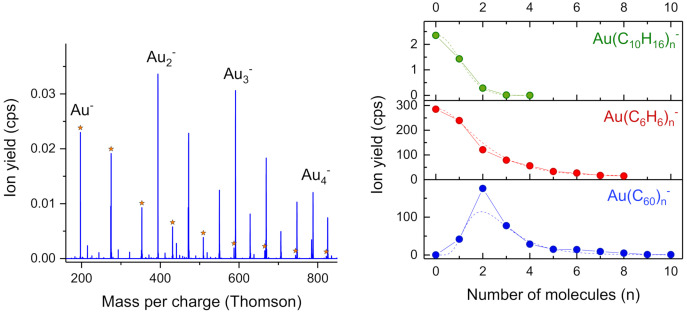
(**Left**): Mass spectrum of negatively charged ions ejected from large HNDs doped with gold and benzene upon irradiation with 22 eV electrons. The Au(C_6_H_6_)_n_^−^ progression is marked with an asterisk. (**Right**): Ligand progression for the anionic clusters Au(C_10_H_16_)_n_^−^, Au(C_6_H_6_)_n_^−^ and Au(C_60_)_n_^−^. An enhancement of the bis-ligand complex is only observed for the fullerene C_60_.

The production of anionic complexes is more demanding than that of cations. In the case of mixed gold-benzene clusters the yield of anions is about four orders of magnitude lower than for cations (see Figure 1 and Figure 8). However, no He*_n_*^−^ cluster ions are formed, which makes the assignment of ions less challenging. All mass spectra of anions were obtained at an electron energy of 22 eV as at this value the yield of anions showed a pronounced maximum. This energy enables electronic excitation of a helium atom and the formation of a low-energy electron inside the HNDs. The inelastically scattered electron will polarize neighboring He atoms and dopants. Ion induced dipole interaction will pull the electron towards the most attractive polarized species. For pristine HNDs this is He* with its huge polarizability of 44.6 Å^3^ [56] (this value is 200 times larger than the polarizability of ground state He and almost comparable to C_60_ which has a value of 76.5 Å^3^ [57]) and in the case of doped HNDs it might be the dopant, depending on its polarizability of the dopant and the relative distance of the electron to the dopant and the nearest He*. Mauracher et al. [58] observed that attachment of the inelastically scattered electron to He* leads at an electron energy of 22 eV to efficient formation of He*^−^. This metastable anion has a potential energy of 19.2 eV and the possibility to form, upon interaction with neighboring species, cations [59], anions [60,61], and dianions [62].

The prominent peaks in the mass spectrum shown in Figure 8 are primarily composed of Au_m_(C_6_H_6_)_n_^−^. Marked with an asterisk is the Au(C_6_H_6_)_n_^−^ series. The size distribution of these ions is plotted in the center right of Figure 8. Both, benzene and adamantane (C_10_H_16_, top) show negligible deviations from log-normal distributions that were fitted to the cluster size distributions (dashed lines). The absence of magic numbers indicates little fragmentation (i.e., monomer loss) after the ionization event or the absence of a particularly strongly bound anionic bis-ligand complex LAuL^−^ for these ligands. However, several studies indicate high binding energies for anionic bis-ligand complexes with gold in sandwich-like structures [41,42,48]. Only C_60_ as a ligand exhibits a magic LAuL^−^ complex, as already reported by Goulart et al. [13]. The high electron affinity of C_60_ and its clusters with gold provide several eV of energy that is sufficient to evaporate some fullerenes. In combination with the exceptionally strong binding of the anionic bis-ligand complex, cluster anions containing one gold atom and more than two C_60_ are likely to dissociate whereas C_60_AuC_60_^−^ has a higher probability to survive and being quenched by the helium matrix. Surprisingly, the enhancement factor for the anionic bis-ligand structure of C_60_ with gold is only 1.54 compared to a factor of 10.44 for C_60_AuC_60_^+^. A tentative explanation for the weak or missing enhancement of the anionic bis-ligand structures is a less energetic ionization mechanism compared to cations, i.e., charge transfer from an initially formed He^+^. Direct attachment of an inelastically scattered electron would be such a non-energetic mechanism, whereas electron transfer from an intermediately formed He*^−^ is expected to additionally deposit 19.2 eV of potential energy into the dopant cluster. Therefore, the formation of cationic fragments is more likely which might explain the low yield for anions compared to cations due to chemical reactions between the ligands and gold.

## 3. Materials and Methods

### 3.1. Experimental Methods

**Figure 9:**
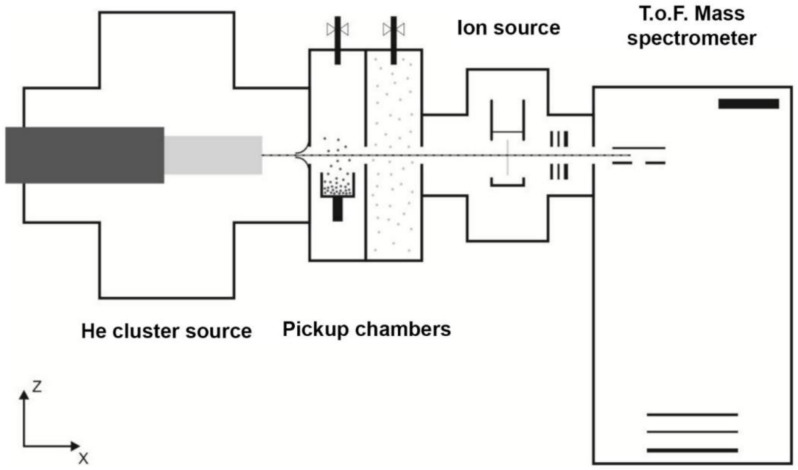
Schematic illustration of the experimental setup ClusTOF. On the left side the helium droplets source, where HNDs are produced via supersonic expansion of precooled He gas into ultrahigh vacuum. This is followed by two differentially pumped sections used to dope the HNDs via collisions with atoms or molecules in the gas phase. Depending on the different samples these atoms or molecules can be evaporated via resistively heated ovens or introduced into the chamber via the gas inlets on the top lid. These doped HNDs are ionized with a crossed beam electron impact ion source placed between the pickup chambers and the time-of-flight mass spectrometer. Low mass ions which were ejected from the HNDs during the ionization are guided into the extraction region of the reflectron time-of-flight mass spectrometer shown on the right side.

The experiments utilize the ultracold environment of helium nanodroplets (HND) as a nano matrix for the formation of cold, mixed complexes with gold as described in [10,11,12,13,14,16,51,63]. The experimental setup which is schematically shown in Figure 9 has been described previously in [64]. HNDs are produced via supersonic expansion of precooled (9–10 K) and pressurized (20–30 bar) He (purity 99.9999%) through a 5 µm pinhole nozzle into ultrahigh vacuum. With these stagnation conditions an average helium droplet size of a few millions He atoms is expected [65,66].

About 1 cm in front of the nozzle the HNDs beam passes a conical skimmer with an aperture of 0.8 mm. This is followed by two differentially pumped chambers used to dope the HNDs by sequential collisions with gaseous atoms or molecules. Samples with a sufficiently high vapor pressure at room temperature were introduced via gas inlets positioned on the top lid of each pickup chamber, resulting in the capture of dopants along the 10 cm long path through these regions. In the following we refer to the first pickup chamber behind the skimmer as PU1, which is followed by the second pickup chamber PU2. The less volatile samples imidazole, dicyclopentadiene, and adamantane were evaporated in a heated container connected via a heated gas line to a resistively heated oven in the center of PU1. The gold oven was placed in PU2 for all the measurements analyzed here. This high temperature evaporation source designed similar to the one used in [20], can reach temperatures of about 1530 K and is well suited for the evaporation of metals. C_60_ was vaporized directly in a heated oven located in PU1. The neutral HNDs passed all ovens via 2 mm diameter openings in the walls and picked up the respective vaporized dopants.

The doped HNDs are then ionized by a Nier-type electron impact ion source, placed in a differentially pumped chamber after the pickup region. For the optimization of the respective signals the electron energy can be varied between 0 and 150 eV (the exact conditions of the HND source, the pickup parameters and the ion source settings are summarized in Table 3). Upon electron ionization low mass ions are ejected from the HNDs and electrostatic fields are used to extract these ions towards an ion guide. This einzel lens stack is used to focus the ions into the extraction region of a commercial high resolution reflectron time-of-flight mass spectrometer (TOF-MS) (H-TOF, Tofwerk AG, Thun, Switzerland). With this TOF-MS a resolution *m/*Δ*m* = 5000 could be reached.

The results were often obtained running several distinct measurements under slightly different experimental conditions to optimize the yield of product ions of interest, i.e., ions containing one gold atom complexed with several ligands. The ion yield of the recorded mass spectra are extracted by the software IsotopeFit [67], which takes into consideration all the isotopic patterns and deconvolutes clusters with the same nominal mass or other overlapping features.

### 3.2. Computational Methods

To highlight the behavior of bis-ligand complexes containing gold we have performed quantum chemical structure calculations of C_5_H_6_ and C_6_H_6_ dimers with Au^+^. The calculations were performed using the Gaussian 16 software package [68] and applies the same methods used in ref. [11] to calculate the structures of mixed gold-imidazole clusters. The calculations used the M06 DFT functional by Zhao and Truhlar [69] together with the def2-TZVP basis set [70,71]. For the Au atoms, the core potential defined in the def2-TZVP basis set was used to describe relativistic inner-shell electrons. Dispersion interactions, which are the main force between the aromatic molecules in our systems, was implemented using Grimme’s D3 model [72]. A vibrational frequency analysis was performed on the optimized structures to ensure that real potential energy minima were obtained and for determining the zero-point corrections of the calculated binding energies

## 4. Conclusions

Mass spectra of cations formed upon electron ionization of HNDs doped with gold and another dopant L (including the rare gases Ne, Ar, Kr and Xe as well as 12 molecules ranging from H_2_ to C_60_) exhibit magic numbers for the bis-ligand complex AuL_2_^+^, most likely in a sandwich-like arrangement. In addition, also the anionic species C_60_AuC_60_^−^ is enhanced compared to the other cluster sizes of the type Au(C_60_)*_n_*^−^. The only exceptions where the bis-ligand structure is not enhanced are anionic clusters containing one gold atom and adamantane or benzene, and cationic clusters of the form AuHe*_n_*^+^. In the case of the anions, we explain the lack of a magic number at AuL_2_ by the absence of ligand loss due to a combination of low excess energy upon anion formation (low electron affinity of the clusters and attachment of an initially formed electron bubble) as well as sufficiently rapid quenching by energy dissipation into the surrounding He matrix before ejection of the anionic complexes. In the case of cationic complexes AuHe*_n_*^+^ the weak binding energy of He ligands to a gold cation is expected to be the reason. The relatively small enhancement in the case of neon for AuNe_2_^+^ supports this explanation, although NeAuNe^+^ was reported to be particularly stable [14]. For some diatomic or dimeric ligands, the presence of gold in the clusters has a significant influence on the relative abundance of non-stoichiometric clusters. While a single Au atom suppresses intramolecular fragmentation of hydrogen molecules by a factor 16, it increases intramolecular fragmentation of oxygen by more than a factor 2. 

## Figures and Tables

**Table 1:** The ratio of the measured ion yield of AuL_2_^+^ (L = ligand) and the corresponding value of the function fitted to the distribution of AuL_n_^+^ is a measure for the relative enhancement of the bis-ligand complex.

Ligand	Enhancement Factor of Bis-Ligand
Anions	
C_60_	1.54
C_6_H_6_	0.82
C_10_H_16_	1.02
Cations	
H_2_O	2.3
CO_2_	1.27
SF_6_	1.72
Xe	2.03
Kr	1.57
Ar	1.66
Ne	1.37
He	1.06
P_2_	1.47
I_2_	1.63
O_2_	1.64
N_2_	2.01
H_2_	3.85
C_6_H_6_	2.19
C_10_H_16_	2.06
C_3_H_4_N_2_	5.29
C_60_	10.44

**Table 2:** Ratio of the yield of stoichiometric to non-stoichiometric ligand gold complexes (Ratio with gold) compared to the ratio of cluster ions of the same molecules without gold (Ratio pristine). The effect on the fragmentation of ligands by the presence of a single gold atom in cluster ions was determined by the ratio of the values listed in column 3 (Ratio pristine) and 2 (Ratio with gold). A value larger than one corresponds to enhanced fragmentation of ligands when gold is present in the cluster ions. (The results for phosphorous are possibly affected by thermal decomposition in the vapor phase, see text).

Ligand	Ratio with Gold	Ratio Pristine	Effect of Gold
C_10_H_12_	1.46	6.73	4.61
P_2_	2.59	2.31	0.89
I_2_	1.29	1.37	1.06
O_2_	1.93	5.16	2.67
N_2_	60.03	97.6	1.63
H_2_	0.82	0.05	0.06

**Table 3:** The experimental conditions for the different measurements showing the temperature (T_He_) and pressure (p_He_) of the helium cluster source, the pressure in the first and second pickup cell (PU1 and PU2, respectively), the heating power of the gold oven, and the energy and current of the ionizing electrons (E_el_ and I_el_, respectively).

Ligand	T_He_ (K)	p_He_ (MPa)	PU	T_oven_ (K)	p_PU1_ (mPa)	P_oven_ Au (W)	p_PU2_ (mPa)	E_el_ (eV)	I_el_ (µA)
C_3_H_4_N_2_, Imidazole	9.7	2.5	Gas inlet into oven PU1	359	1.21	118	0.34	110	82
C_10_H_12_, di-cyclo-pentadiene	10	3	Gas inlet into oven PU1		0.55	144	0.28	88	160
C_6_H_6_	9.6	2.7	Gas inlet PU2		1.1	128	2.81	93	247
C_60_	9.5	2.25	Oven PU1	590	0.92	106	0.29	73	88
C_10_H_16_, adamantane	9.8	2.3	Gas inlet PU1		1	100–166		70	255
I_2_	9.3	2.1	Gas inlet PU1		6.9	144	0.22	142	118
O_2_	9.4	2.5	Gas inlet PU1		1.35	120	0.47	95	110
N_2_	9.7	2.6	Gas inlet PU1		1.43	120	0.48	85	97
H_2_	9.5	2.6	Gas inlet PU1		1.2	120	0.49	85	93
P_2_	9.6	2.4	from Au oven		9.4	60	0.55	80	33
Xe	9.6	2.2	Gas inlet PU1		1	113	0.34	63.5	117
Kr	9.5	2.4	Gas inlet PU1		1	116	0.38	91.2	101
Ar	9.4	2.1	Gas inlet PU1		1	114	0.36	52	127
Ne	9.7	2.2	Gas inlet PU2		1	114	2.2	57	133
He	9.4	2.25			0.96	104	0.31	55	109
SF_6_	9.4	2.4	Gas inlet PU1		1	117	0.35	115	74
CO_2_	9.7	2.5	Gas inlet PU1		1.3	120	0.41	85	97
H_2_O	9.4	2.1	Gas inlet PU2		0.88	189	-	90	51

## Data Availability

Data is contained within the article/on demand to the authors.

## Sample Availability

Samples of the compounds are not available.
